# Pilot Study: FSHR Expression in Neuroendocrine Tumors of the Appendix

**DOI:** 10.3390/jcm12155086

**Published:** 2023-08-02

**Authors:** Dariusz Starzyński, Sylwia Rzeszotek, Agnieszka Kolasa, Marta Grabowska, Barbara Wiszniewska, Aleksandra Kudrymska, Katarzyna Karpińska, Aleksandra Tołoczko-Grabarek, Agnieszka Janiec, Aleksandra Myszka, Paweł Rynio, Anhelli Syrenicz, Elżbieta Sowińska-Przepiera

**Affiliations:** 1 Department of Endocrinology, Metabolic and Internal Diseases, Faculty of Medicine and Dentistry, Pomeranian Medical University in Szczecin, Unii Lubelskiej 1, 70-252 Szczecin, Poland; starzynskimd@gmail.com (D.S.); agnieszka.janiec@pum.edu.pl (A.J.); aleksandra.myszka@pum.edu.pl (A.M.); anhelli.syrenicz@pum.edu.pl (A.S.); elasowprzep@wp.pl (E.S.-P.); 2 Department of Histology and Embryology, Faculty of Medicine and Dentistry, Pomeranian Medical University in Szczecin, Powstańców Wlkp. 72, 70-111 Szczecin, Poland; agnieszka.kolasa@pum.edu.pl (A.K.); barbara.wiszniewska@pum.edu.pl (B.W.); 3 Department of Histology and Developmental Biology, Faculty of Health Sciences, Pomeranian Medical University, Żołnierska 48, 71-210 Szczecin, Poland; marta.grabowska@pum.edu.pl; 4 Department of Pathomorphology, Faculty of Medicine and Dentistry, Pomeranian Medical University in Szczecin, Unii Lubelskiej 1, 70-252 Szczecin, Poland; olaqdr@wp.pl (A.K.); kkarp7275@gmail.com (K.K.); 5 Department of Genetics and Pathomorphology, Faculty of Medicine and Dentistry, Pomeranian Medical University in Szczecin, Powstańców Wlkp. 72, 70-111 Szczecin, Poland; aleksandra.toloczko.grabarek@pum.edu.pl; 6 Department of Vascular Surgery, General Surgery and Angiology, Faculty of Medicine and Dentistry, Pomeranian Medical University in Szczecin, 70-111 Szczecin, Poland; ryniopawel@gmail.com

**Keywords:** appendix neuroendocrine neoplasm, ANEN, FSHR, endothelial cells

## Abstract

Appendix neuroendocrine neoplasm (ANEN) treatment is based on tumor size and proliferation markers. Recently, the role of the follicle-stimulating hormone receptor (FSHR) from the clinical perspective has also been increasingly discussed. The FSHR is expressed in the endothelial cells of both intratumoral and peritumoral blood vessels, where it contributes to neoangiogenesis and blood vessel remodeling. FSHR expression is associated with a range of tumor types, such as gastrointestinal tumors, and it is not detected in healthy tissues located more than 10 mm from the tumor site or in tumor lymphatics. In this study, we evaluated the expression of FSHR and CD31 in the blood vessels of ANENs in females and males with confirmed histopathology. We conducted a quantitative analysis of the immunohistochemical reactions and found a higher number of microvessels in the mucosa and submucosa of neuroendocrine tumors in the appendix. A higher level of FSHR expression was observed in women. Future research should consider whether an elevated number of blood vessels along with a strong pattern of FSHR expression may influence future treatment strategies.

## 1. Introduction

An appendix neuroendocrine neoplasm (ANEN) is a rare tumor that is typically discovered incidentally following an appendectomy. Treatment for ANEN depends on the tumor size and proliferation markers. Larger tumors have a higher risk of lymphatic spread, while small tumors pose no such risk [1]. Unlike other neuroendocrine neoplasms (NENs), ANENs tend to occur in younger patients, usually between the ages of 15 and 29 or 32 and 42, whereas NENs are typically diagnosed in people in their sixth decade of life or older [2]. Additionally, there is a slight female predominance in ANEN cases (60.5%) [3,4]. Treatment of patients with well-differentiated ANENs at an early stage of the disease is important due to the possibility of complete resection of the appendix tumor, thus resulting in complete recovery of the patient. Highly differentiated ANENs are characterized by a slow growth rate, but as the time to diagnosis and effective treatment increases, the risk of tumor growth, local infiltration, and distant metastases increases. A greater advancement of ANENs is associated with a worse prognosis for the patient, more invasive methods of treatment, and lower effectiveness thereof. Nevertheless, treatment is important at every stage of the disease [5,6].

In the laboratory diagnosis of ANENs, the determination of the concentration of chromogranin A (CgA) is particularly helpful in assessing the response to treatment and detecting progression and recurrence at an early stage. Other non-specific markers are neuron-specific enolase (NSE) and pancreatic polypeptide (PP), but they are characterized by lower sensitivity and specificity than those of CgA. A screening test may also allow the determination of the daily excretion of 5-hydroxyindole acetic acid (5-HIAA), which is a serotonin metabolite, in urine [7]. The imaging diagnostics of ANENs use computed tomography (CT) or magnetic resonance imaging (MR) of the abdomen [8], colonoscopy [9], and nuclear medicine techniques, such as PET/CT with a Ga68-labeled somatostatin analog [10].

In neuroendocrine neoplasms, there is the so-called paradox of neuroendocrine tumors, which concerns the fact that dense vascularity is an indicator of high differentiation of a tumor and not its aggressiveness. Thus, the greatest vascularization occurs in NENs with the highest degree of differentiation, which stands in opposition to other types of epithelial tumors [11]. In the work of Yazdani et al., the density of microvessels in neuroendocrine tumors was assessed by using markers of endothelial cells, e.g., CD31 and vasohibin-1 (VASH-1). It was confirmed that the VASH-1/CD31 ratio can be a reliable indicator for the assessment of neovascularization in neuroendocrine tumors [12]. This prompted us to use the CD31 marker as an objective indicator for the assessment of ANEN vascularization in our study.

The fundamental mechanism of follicle-stimulating hormone (FSH) on human fertility is a direct impact on the gonads. Recent scientific reports have expanded our understanding of the pleiotropic effects of FSH and its impact on cells through its four FSH receptor (FSHR) isoforms. FSHR is a crucial signaling element that is present in both males and females and is expressed in healthy reproductive tissues, osteoclasts, chondrocytes, adipocytes, some parts of the brain, and minimally in the endothelial cells of gonadal blood vessels. It is noteworthy that FSHR may also be temporarily expressed in tissue-/organ-specific progenitor/stem cells and then may disappear during cellular differentiation, similarly to Oct-4 [13,14,15,16]. Importantly, FSHRs are present on cancer cells that originate from rapidly dividing stem/progenitor cells. This observation explains the extragonadal occurrence of FSHR in tumor cells from various organs [17,18]. In contrast, mature somatic cells surrounding the tumor do not possess FSHR.

In 2010, it was reported that FSHR can be expressed on the endothelial cells of the intratumoral and peritumoral blood vessels associated with different types of tumors, such as gastrointestinal tumors [19]. In prostate cancer, endothelial cells expressing FSHR were located at the periphery of the tumors and extended a few millimeters both inside and outside the tumors in apparently normal tissue [20]. It has been found that in numerous tumors, FSHR expression stimulates proliferation and metastasis, while vascular endothelial FSHR promotes tumor angiogenesis and blood vessel remodeling [21]. Notably, not only FSH, but also other pituitary gonadotrophins are potent mitogens and chemoattractants, and they can alter the adhesion potential of some tumor cells [22].

Based on these observations, we conducted an evaluation of FSHR and CD31 expression in the blood vessels of ANENs in both males and females. Our results can enhance our understanding of the role of the tumor vasculature microenvironment and potentially open up new diagnostic and therapeutic strategies. Our study revealed notable differences in FSHR expression in ANENs between females and males.

## 2. Materials and Methods

### 2.1. Patient Samples

**Figure 1:**
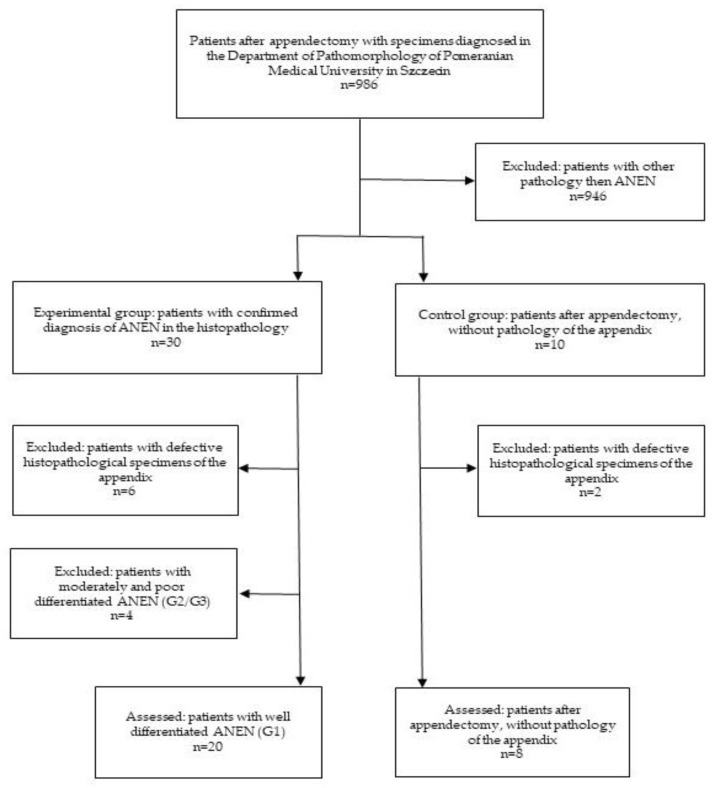
Diagram showing the selection criteria for the samples.

**Figure 2:**
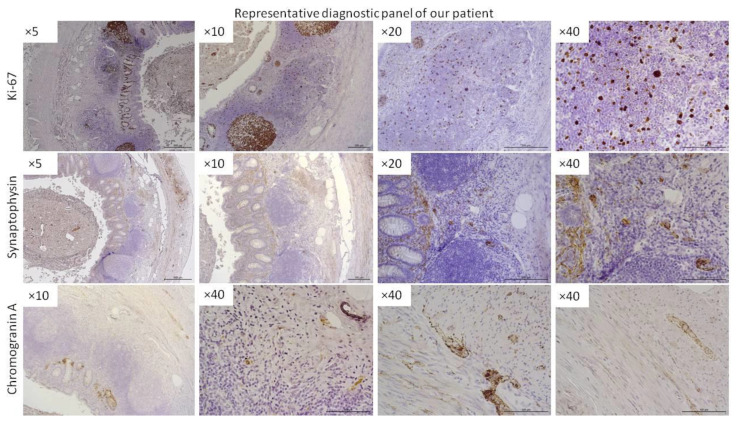
Representative panel of immunohistochemical diagnostics of ANEN patients. More representative data are provided in the Supplementary Data. Stainings for Ki-67, synaptophysin, and chromogranin A were performed. The magnification of the lens is given (Leica DM5000B, Wetzlar, Germany).

This study was approved by the Pomeranian Medical University Bioethics Committee and was conducted according to the principles of the Declaration of Helsinki. Tissue samples were obtained from patients with histopathologically confirmed ANENs, and controls were obtained from patients following an appendectomy after obtaining written consent. The sampling scheme is summarized in Figure 1. A representative diagnostic panel is shown in Figure 2.

All patients underwent subsequent treatment according to the current guidelines. The staging of the disease according to the UICC (Union for International Cancer Control)/AJCC (American Joint Committee on Cancer) and WHO (World Health Organization) classifications, Ki67 level, tumor size, and tumor infiltration of the appendix layers were recorded and are presented in Table 1.

### 2.2. Tissue Preparation

The dissected appendices were fixed in 10% formalin for at least 24 h and then washed with absolute ethanol (3 times over 3 h), absolute ethanol with xylene (1:1) (twice over 1 h), and xylene (3 times over 20 min). Then, after 3 h of saturation of the tissues in liquid paraffin, the samples were embedded in paraffin blocks. By using a microtome (Microm HM340E, Thermo Fisher Scientific, Walldorf, Germany), 3–5 μm serial sections were taken and placed on poly-l-lysine microscope slides (Thermo Scientific, Leicestershire, UK; cat. no. J2800AMNZ). The sections of the appendices were deparaffinized in xylene, rehydrated in decreasing concentrations of ethanol, and then used for immunohistochemical (IHC) reactions.

### 2.3. Immunohistochemical Reactions

In order to expose the epitopes for the IHC procedure, the deparaffinized and rehydrated sections were boiled in Target Retrieval Solution (DakoCytomation, Glostrup, Denmark, S2369) in a microwave oven (twice 700 W for 5 min). Once cooled and washed with PBS, the endogenous peroxidase was blocked by using a 3% solution of perhydrol in methanol, and then the slides were incubated overnight at 4 °C with primary antibodies: monoclonal anti-CD31 (abcam, Cambridge, UK, ab231436; final dilution 8 μg/mL) and polyclonal anti-FSHR (abcam, Cambridge, UK, ab150557; final dilution 1:50) in antibody diluent with background-reducing components (Dako, Santa Clara, CA, USA, S3022). To visualize the antigen–antibody complexes, a Dako LSAB+System-HRP was used (DakoCytomation, Glostrup, Denmark, K0679) based on the reaction of avidin–biotin–horseradish peroxidase with DAB as a chromogen according to the included staining procedure instructions. Sections were washed in distilled H_2_O and counterstained with hematoxylin. For the negative control, specimens were processed in the absence of primary antibodies. Positive staining was determined microscopically (Leica DM5000B, Wetzlar, Germany) through visual identification of brown pigmentation [23].

### 2.4. Quantitative Analysis of Immunohistochemistry

**Figure 3:**
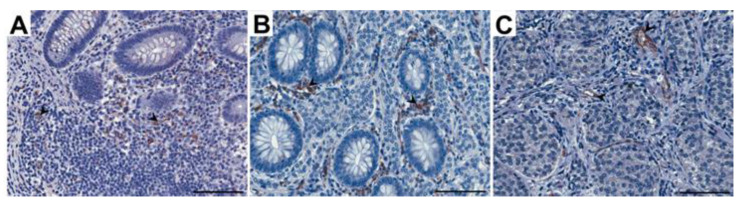
Representative light micrographs showing CD31 immunoexpression in endothelial cells of vessels in the appendix: control group (**A**) and patients with neuroendocrine neoplasms in the mucosa (**B**) and submucosa (**C**). Scale bar: 50 µm. Black arrows show blood vessels.

All slides obtained under the immunostaining of FSHR and CD31 were scanned at 400× magnification (resolution of 0.25 μm/pixel) by using a ScanScope AT2 scanner (Figure 2 and Figure 3; Leica Microsystems, Wetzlar, Germany). In order to minimize focus problems, the scanner was preconfigured. The ImageScope viewer software (Aperio Technologies, Inc., Vista, CA, USA) provided digital images for examination on a computer screen. For the quantitative analysis of FSHR expression in the plasma membranes of the neuroendocrine tumor cells in particular layers of the appendix, the membrane v9 algorithm (Aperio Technologies, Inc., Vista, CA, USA) was used. The percentages of FSHR-positive cells with weak, moderate, and strong immunostaining in the plasma membranes of tumoral cells were determined. The percentage of FSHR-positive cells was counted in a total of 110 random fields in particular layers of the appendix, with an average area of 0.04 mm^2^ for mucosa and submucosa, 0.03 mm^2^ for muscle layers, and 0.02 mm^2^ for serosa. For a detailed analysis of the CD31 expression in the particular layers of the appendix, the microvessel analysis v1 algorithm (Aperio Technologies, Inc., Vista, CA, USA) was used. The algorithm automatically classified the staining as weak, moderate, or strong depending on the optical density ranges for a given level of immunoexpression. The microvessel density, mean vessel area, and mean vessel perimeter were calculated. The quantitative data for CD31 were assessed in a total of 38 random high-power fields in particular layers of the appendix, with an average area of 1.03 mm^2^ for mucosa and submucosa, 0.97 mm^2^ for muscle layers, and 0.81 mm^2^ for serosa.

### 2.5. Statistical Analysis

Statistical analyses were performed with the Statistica v13.1 software (StatSoft, Krakow, Poland). The normality of the distribution for the quantitative values was evaluated by using a Shapiro–Wilk test, and as most of the results deviated from a normal distribution, a nonparametric test was used for further analysis. To evaluate the differences between the groups, nonparametric Mann–Whitney U-tests and Kruskal–Wallis tests with Dunn’s multiple comparison for post hoc analysis were used. The level of statistical significance was *p* ≤ 0.05.

## 3. Results

### 3.1. CD31 Immunoexpression

The appendices of both the healthy patients (control group) and those with ANENs showed immunoexpression of CD31 that was visible as brown-stained endothelial cells of the vessels (Figure 3). A CD31-positive vascular endothelium was revealed in patients with ANENs in the peritumoral and intratumoral vessels. The number of vessels per unit area (microvessel density) in the mucosa and submucosa of the appendix in patients with ANENs was statistically significantly higher (*p* < 0.02) than that in the control group, while in the muscle layer and serosa, there was no statistical significance. In patients with ANENs, the mean vessel area and mean vessel perimeter in all layers of the appendix showed no statistically significant differences compared to the controls (Table 2).

Microvessel density is a well-established prognostic marker in many types of tumors [24]. Preoperative assessment of microvessel density in nonfunctioning neuroendocrine tumors of the pancreas showed that low microvessel density is a marker of tumor aggressiveness in these patients [25].

### 3.2. Pattern of FSHR Immunoexpression in ANENs without Division into Women and Men

**Figure 4:**
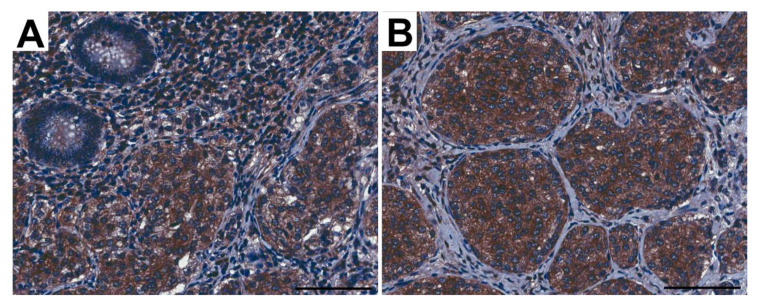
Representative light micrographs showing FSHR immunoexpression in tumoral cells in the appendix from patients with neuroendocrine neoplasms in the mucosa (**A**) and submucosa (**B**). Scale bar: 50 µm.

**Figure 5:**
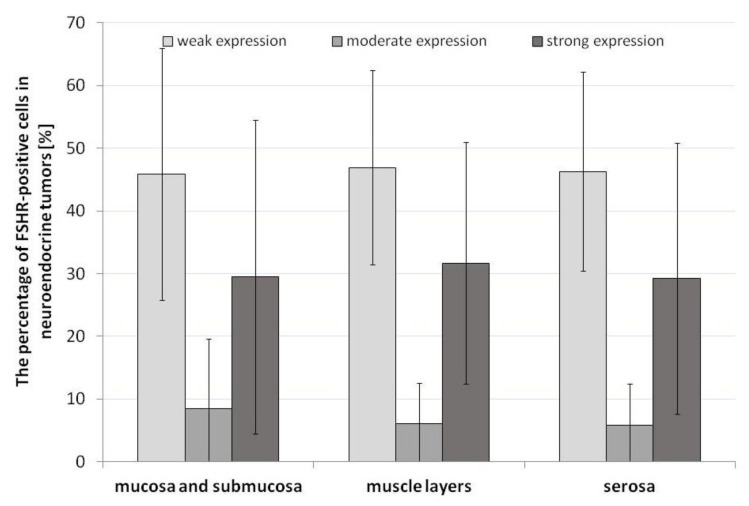
Percentage of FSHR-positive cells in the plasma membranes of tumoral cells of the appendix in patients with neuroendocrine neoplasms. Lack of statistical differences.

The immunohistochemical analysis revealed the immunoexpression of FSHR in the appendix of patients with ANENs that was visible as brown staining of the plasma membranes and the cytoplasm of tumoral cells, as well as the endothelial cells of vessels (Figure 4). The percentages of FSHR-positive cells with weak, moderate, or strong expression in neuroendocrine tumors showed no statistical differences between the layers of the appendix (Figure 5).

### 3.3. Pattern of FSHR Immunoexpression in ANENs by Gender

Due to the fact that FSH and, thus, FSHR are strongly related to gender, it was necessary to assess the strength of FSHR expression in specific layers while taking the gender of patients into account.

#### 3.3.1. Mucosa and Submucosa

**Figure 6:**
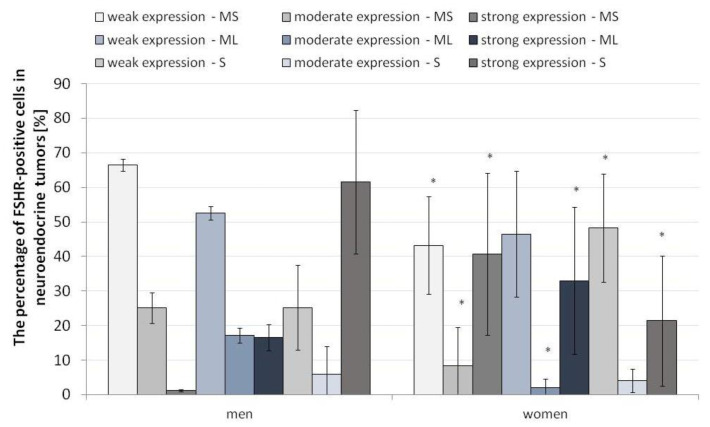
Percentage of FSHR-positive cells in the appendix in neuroendocrine neoplasms by gender. The division into strong, moderate and weak FSHR expression is marked. * *p* ≤ 0.05. Asterisks show differences in the level of FSHR-positive cells in males and females in specific layers of ANENs, including the strength of the signal (weak, moderate, strong). N = 20 females, n = 5 males. MS—mucosa and submucosa, ML—muscle layers, S—serosa.

The expression pattern of FSHR in the different layers of the neuroendocrine tumors revealed statistically significant differences between men and women. In men, weak and moderate expression of FSHR was higher in the mucosa and submucosa of the ANEN, while women had higher levels of strong FSHR expression (Figure 6). It is noteworthy that gender plays a crucial role in FSHR expression.

#### 3.3.2. Muscle Layers

In the muscle membrane layer, the differences were only visible for moderate and strong expression. The pattern of moderate FSHR expression in the muscular membrane was higher in men than in women, while strong expression, as in the mucosa and submucosa, was higher in the women (Figure 6).

#### 3.3.3. Serosa

In the serosa, we observed converse tendencies to the other layers of the appendix wall. Low FSHR expression was dominant in the women, and strong expression was more pronounced in the men. The differences in the pattern of moderate expression of FSHR were not statistically significant (Figure 6).

#### 3.3.4. CD31 and FSHR Co-Expression in Endothelial Cells of Capillaries

**Figure 7:**
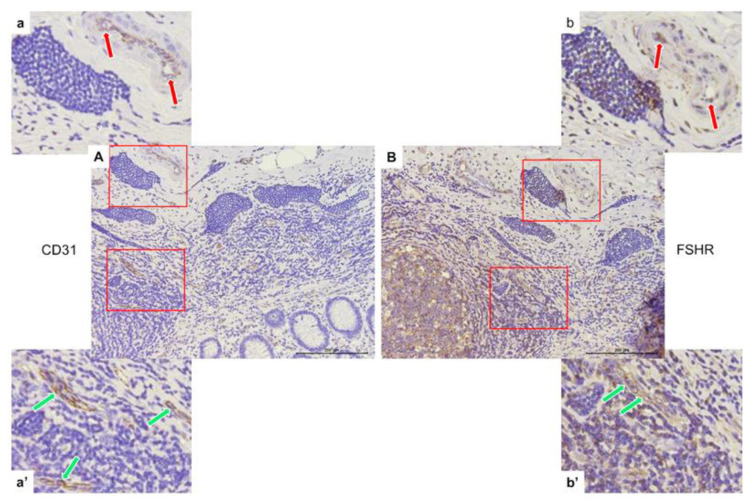
Representative light micrographs showing CD31 (**A**,**a**,**a’**) and FSHR (**B**,**b**,**b’**) co-expression in peritumoral (red arrows) and intratumoral (green arrows) endothelial cells of capillaries in the appendices of patients with neuroendocrine neoplasms in the mucosa. Scale bar: 200 µm.

The same area of appendix tissue (Figure 7) from different immunostaining slides was selected to show the co-expression of CD31 (A, a, a’) and FSHR (B, b, b’) in endothelial cells of capillaries growing in neuroendocrine tumors (green arrows) and into peritumoral (red arrows) areas. 

## 4. Discussion

Our work revealed that FSHR expression was detected in the endothelial cells involved in angiogenesis and vascular remodeling in ANENs, indicating its potential role in these processes. Moreover, we observed increased microvessel density in the mucous and submucous layers of ANENs in the appendix.

Selective FSHR expression on the surface of blood vessels has been observed in various types of cancers. Radu et al. [20] conducted a study involving 1336 patients with different tumors, showing FSHR expression on endothelial cells located at the periphery of tumors in a layer that was about 10 mm thick both inside and outside the tumor, irrespective of the tumor stage. Notably, that study showed that FSHR expression occurred exclusively on the endothelial cells of blood vessels, not lymphatic vessels, which may be associated with the spread of the tumor through the bloodstream [20].

Interestingly, Siraj et al. [26] observed increased FSHR expression in blood vessels both inside and outside most metastatic lesions. In breast, lung, colon, and kidney cancer, the density of FSHR expression in intra-metastatic vessels was constant up to 7 mm and did not differ from the peri-metastatic vessels, with the exception of renal cancer metastasis, which had an FSHR expression density that was three times higher outside the tumor compared to the interior [16,27]. These findings suggest that a higher density of FSHR expression in the blood vessels around a tumor may be indicative of a more aggressive tumor.

The functions of FSHR vary between epithelial cells and endothelial cells in tumor blood vessels. Within the epithelium, FSHR plays a role in the proliferation, migration, and invasion of tumor cells. In contrast within endothelial cells, FSHR is implicated in neoangiogenesis and vessel remodeling. The peripheral positioning of vessels expressing FSHR in a tumor presents a potential target for treatment. By combining the extracellular domain of human tissue factor with anti-FSHR antibodies, the coagulation cascade may be activated, resulting in thrombus formation, ischemia, and cancer cell death. Nevertheless, to circumvent severe complications, it is crucial to determine a safe concentration of the drug that predominantly targets the tumor [21].

Continued investigation into FSHR expression in various tumor types, such as low-grade T1 and metastatic tumors, may facilitate the development of tumor-specific fluorescent agents to assess surgical margins. Identifying these boundaries under standard conditions is challenging due to the microinfiltration of healthy tissues by tumors [28]. 

The use of fluorescently labeled FSHR agonists remains in the experimental phase, necessitating further examination of their toxicity, pharmacokinetics, and pharmacodynamics. Nevertheless, FSHR is a highly promising tool in the future fight against cancer. Elevated FSHR expression on tumor endothelial cells could serve as a diagnostic factor and predictor of response to angiogenesis-inhibiting treatment, particularly in tumors with increased angiogenesis and growth factor association [29]. 

The reports of Pawlikowski et al. [19,30] on the expression of FSHR on thyroid cancer cells and invasive pituitary adenomas suggest that FSHR may indicate tumor aggressiveness. In ANENs, combining FSHR assessment with the Ki-67 proliferation index may enhance patient monitoring and facilitate therapeutic decisions.

Based on the NANETS (North American Neuroendocrine Tumor Society) and ENETS (European Neuroendocrine Tumor Society) guidelines, patients with G1/G2 ANENs with a diameter of >20 mm, G3, and NEC should undergo right hemicolectomy, while G1/G2 tumors between 10 and 20 mm in diameter located at the base of the appendix and with positive surgical margins, metastases to lymph nodes, angioinvasion, infiltration of the mesoappendix of >3 mm, and Ki-67 > 3%, a hemicolectomy should be considered [6,31,32]. Studies using FSHR expression to determine tumor infiltration depth indicated successful tumor resections for lesions that were <20 mm, avoiding a supplementary hemicolectomy without additional risk factors. The multicenter study conducted by Nesti et al. [33] found no impact on long-term survival from right-sided hemicolectomy in patients with ANENs measuring 10–20 mm, casting doubt on the current NANETS and ENETS guidelines for this patient group.

In a study by Siraj et al. [27], 50 patients with advanced clear-cell renal cell carcinoma (RCC) underwent surgery followed by sunitinib therapy. Before initiating sunitinib, the researchers assessed vessel density and FSHR expression levels within these vessels. Patients with higher FSHR expression exhibited better treatment outcomes [27]. Among the approved targeted therapies for pancreatic neuroendocrine tumors is sunitinib [34]. It is conceivable that sunitinib may be employed in the future to treat patients with ANENs that exhibit distinct FSHR expression. 

For pancreatic neuroendocrine tumors, co-expression of FSHR and chromogranin was observed in tumor cells, with no expression of FSHR on the endothelium labeled with von Willebrandt factor. The intensity of FSHR expression did not correlate with histological features of the pancreatic neuroendocrine tumors [35].

Ongoing research explores the potential application of FSHR in diagnosis and future treatment strategies. Positron emission tomography (PET) using an anti-FSHR monoclonal antibody (FSHR-mAb) conjugated with S-2-(4-isothiocyanatobenzyl)-1,4,7-triazacyclononane-1,4,7-triacetic acid (p-SCN-BnNOTA), and a radiotracer (^64^Cu) demonstrated a high affinity to FSHR in ovarian cancers compared to FSHR-negative tumors [36]. Another pre-clinical PET study by Xu et al. [37] also suggested that chemical conjugates of FSHR may be suitable radiotracers for non-invasive imaging of FSHR-expressing tumors.

Currently, PET with ^68^Ga is commonly used for imaging neuroendocrine neoplasms due to the increased expression of somatostatin receptors on tumor cells. New PET tracers using aluminum fluoride and ^64^Cu, which exhibit affinity for FSHR, offer hope for more precise imaging of previously undetectable lesions [32]. 

Epidemiological data suggest a stronger association between the female gender and ANENs compared to the same association for males, which is potentially due to the higher appendectomy rates in younger women [38,39]. Early menopausal transition is associated with increased serum FSH levels in younger women. Although FSH levels are similar in males and females before and during puberty, they differ in adulthood, with women experiencing increased fluctuations. In our study, high FSHR expression was characteristic of women. Serum FSH levels were not determined in the present study, and the average ages of the women and men were 33.8 and 35.6, respectively. 

The present study has several limitations. First, extragonadal expression of FSHR can be controversial, as some authors have suggested that the reliable assessment of tumor samples and control samples requires the use of monoclonal antibodies (which act selectively on a specific isoform of FSHR) instead of the polyclonal antibodies used in our study. In our research, FSHR was evaluated in the appendix in the plasma membranes of tumor cells in patients with neuroendocrine neoplasms, and we observed negative signals from normal control tissues. Secondly, we used polyclonal anti-FSHR antibodies (abcam) that bound to different FSHR isoforms. In light of current knowledge, it is not clear which FSHR isoform plays a key role in the development and progression of neoplasms. According to a study by Bhartiya [10], FSH acts primarily via alternatively spliced FSHR-3 rather than the canonical FSHR-1 on stem/progenitor cells, from which neoplasms may develop. Additionally, discrepancies between FSHR transcript and protein expression levels should be considered. Doroszko et al. [40] failed to detect FSHR transcripts or protein of patients with adrenocortical carcinoma, unlike in an earlier study by Pawlikowski et al. [30], who used IHC alone. Ensuring the specificity and sensitivity of FSHR detection requires tandem RNA and protein measurements [41]. 

## 5. Conclusions

As a considerable proportion of cancers across various locations exhibit increased FSHR expression in endothelial cells, further research should be aimed at developing advanced diagnostic methods that enable precise localization of primary tumors and metastatic lesions, predictive assessment, and accurate determination of the scope of surgical procedures. Another critical aspect is the potential use of FSHR expression in developing targeted anticancer therapies, as demonstrated by Hong [29]. Targeted therapy may yield improved therapeutic outcomes while significantly reducing side effects. The aim of this study was to show that such determinations may have clinical value in the future development of new standards of treatment (e.g., determination of the tissue margin during resection or the use of drugs affecting tissues with FSHR expression) or diagnostics (e.g., new radioisotope tracers used in PET). Currently, due to the lack of standard determination of FSHR in ANENs, there is no direct translation of such determinations into clinical applications. 

Our study, in comparison to other publications, not only identified FSHR immunoexpression in ANENs, but also assessed the patterns of FSHR expression (weak, moderate, and strong). Moreover, we observed an increased number of microvessels in the mucosa and submucosa of neuroendocrine tumors of the appendix compared to the control. Future research should consider whether an elevated number of blood vessels, along with a strong pattern of FSHR expression, influences local recurrence or metastasis.

## Figures and Tables

**Table 1:** Characteristics of the material collected for evaluation: sex and age of the patients, size of the tumor, staging based on the UICC (Union for International Cancer Control)/AJCC (American Joint Committee on Cancer) and WHO (World Health Organization) guidelines, level of Ki-67 expression, mitotic count, levels of synaptophysin, chromogranin A, and FSHR expression, and tumor infiltration of the appendix layers. + shows the presence of tumor infiltration of the specific layers or positive immunoexpression; − shows the lack of tumor invasion in a specific layer or negative immunoexpression.

ANEN No.	Sex	Age	Tumor Size [mm]	Staging (AJCC/UICC 2017)	Grading (WHO 2022)	Ki-67	Mitotic Count [per 2 mm^2^]	Chromogranin A	Synaptophysin	Mucosa Invasion	Submucosa Invasion	Muscular layer Invasion	Serosa Invasion	FSHR
1	F	56	7	pT1	G1	1%	1	+	+	+	+	+	−	+
2	F	10	3	pT1	G1	1%	0.5	+	+	+	+	−	−	+
3	F	13	6	pT1	G1	1%	0.2	+	+	+	+	+	−	+
4	F	64	8	pT1	G1	1%	1	+	+	+	+	+	−	+
5	F	25	3	pT1	G1	2%	1	+	+	+	+	−	−	+
6	M	25	5	pT1	G1	1%	1	+	+	+	+	+	+	+
7	F	52	3	pT1	G1	1%	1	+	+	+	+	−	−	+
8	F	36	9	pT1	G1	2%	0.5	+	+	+	+	−	−	+
9	F	47	11	pT1	G1	1%	1	+	+	+	+	+	+	+
10	F	25	6	pT1	G1	1%	1	+	+	+	+	+	−	+
11	F	18	1	pT1	G1	1%	0.8	+	+	+	+	+	−	+
12	F	20	7	pT1	G1	1%	1	+	+	+	+	+	−	+
13	M	53	9	pT1	G1	1%	1	+	+	+	+	+	−	+
14	F	59	5	pT1	G1	1%	0.5	+	+	+	+	−	−	+
15	F	22	14	pT1	G1	1%	1	+	+	+	+	+	−	+
16	F	21	5	pT1	G1	2%	0.5	+	+	+	+	+	−	+
17	F	21	3	pT1	G1	0.5%	0.1	+	+	+	+	+	−	+
18	M	29	2	pT1	G1	1%	1	+	+	+	+	−	−	+
19	F	30	5	pT1	G1	2%	0.2	+	+	+	+	+	−	+
20	F	56	6	pT1	G1	1%	1	+	+	+	+	+	−	+

**Table 2:** Vessel parameters in the control group and in patients with neuroendocrine neoplasms. Legend: Microvessel density—number of vessels per unit area (μm^2^); NEN—patients with neuroendocrine neoplasms; X ± SD—arithmetical mean ± standard deviation; *—*p* < 0.05 vs. control (Mann–Whitney U test).

Group	Layer of Appendix	Microvessel Density		Mean Vessel Area (µm^2^)		Mean Vessel Perimeter (µm)	
		Median (Range)	X ± SD	Median (Range)	X ± SD	Median (Range)	X ± SD
Control	Mucosa and submucosa	2.3 × 10^−6^ (2.6 × 10^−8^–5.0 × 10^−5^)	1.0 × 10^−5^ ± 1.4 × 10^−5^	104.6 (39.0−320.9)	123.9 ± 73.3	50.8 (28.0−87.2)	50.6 ± 16.6
	Muscle layer	2.8 × 10^−6^ (1.3 × 10^−7^–3.7 × 10^−5^)	6.8 × 10^−6^ ± 9.3 × 10^−6^	81.7 (36.8−172.1)	85.6 ± 33.8	40.8 (15.7−94.0)	45.4 ± 15.2
	Serosa	2.3 × 10^−5^ (8.7 × 10^−7^–2.6 × 10^−4^)	4.7 × 10^−5^ ± 5.9 × 10^−5^	225.5 (17.0−392.7)	240.6 ± 101.0	82.6 (19.0−106.3)	82.4 ± 19.7
ANEN	Mucosa and submucosa	9.1 × 10^−6^ (5.6 × 10^−7^–7.0 × 10^−5^)	1.4 × 10^−5^ * ± 1.5 × 10^−5^	138.3 (34.0−313.0)	144.7 ± 68.3	55.5 (29.5−96.3)	57.5 ± 16.5
	Muscle layer	4.1 × 10^−6^ (1.0 × 10^−6^–2.1 × 10^−4^)	1.2 × 10^−5^ ± 3.5 × 10^−5^	82.9 (21.0−214.6)	93.9 ± 54.9	42.3 (22.0−81.5)	45.4 ± 19.5
	Serosa	4.1 × 10^−5^ (2.6 × 10^−7^–1.8 × 10^−4^)	5.6 × 10^−5^ ± 5.2 × 10^−5^	212.0 (18.0−532.4)	255.5 ± 123.7	80.0 (19.0−123.0)	82.5 ± 25.4

## Data Availability

Access to the data is entirely possible upon request addressed to the authors, who have stored the data in the IT system of the Pomeranian Medical University.

## Supplementary Materials

The following supporting information can be downloaded at: https://www.mdpi.com/article/10.3390/jcm12155086/s1, which includes representative figures of the patients’ diagnostic panels and controls for FSHR from patients without tumors (control group). We can also see that in the blood vessels, there were no positive signals. These are raw data.
