# Supplementary material for: Pilot Study: FSHR Expression in Neuroendocrine Tumors of the Appendix
Source: J Clin Med. 2023 Aug 2;12(15):5086. doi: 10.3390/jcm12155086 (PMC10419379; doi:10.3390/jcm12155086)
Supplement: Supplementary file 1 [file jcm-12-05086-s001.zip › dianostic panel_Supplementary data.pdf]

N2XXXX_6_4_Chromogranin A

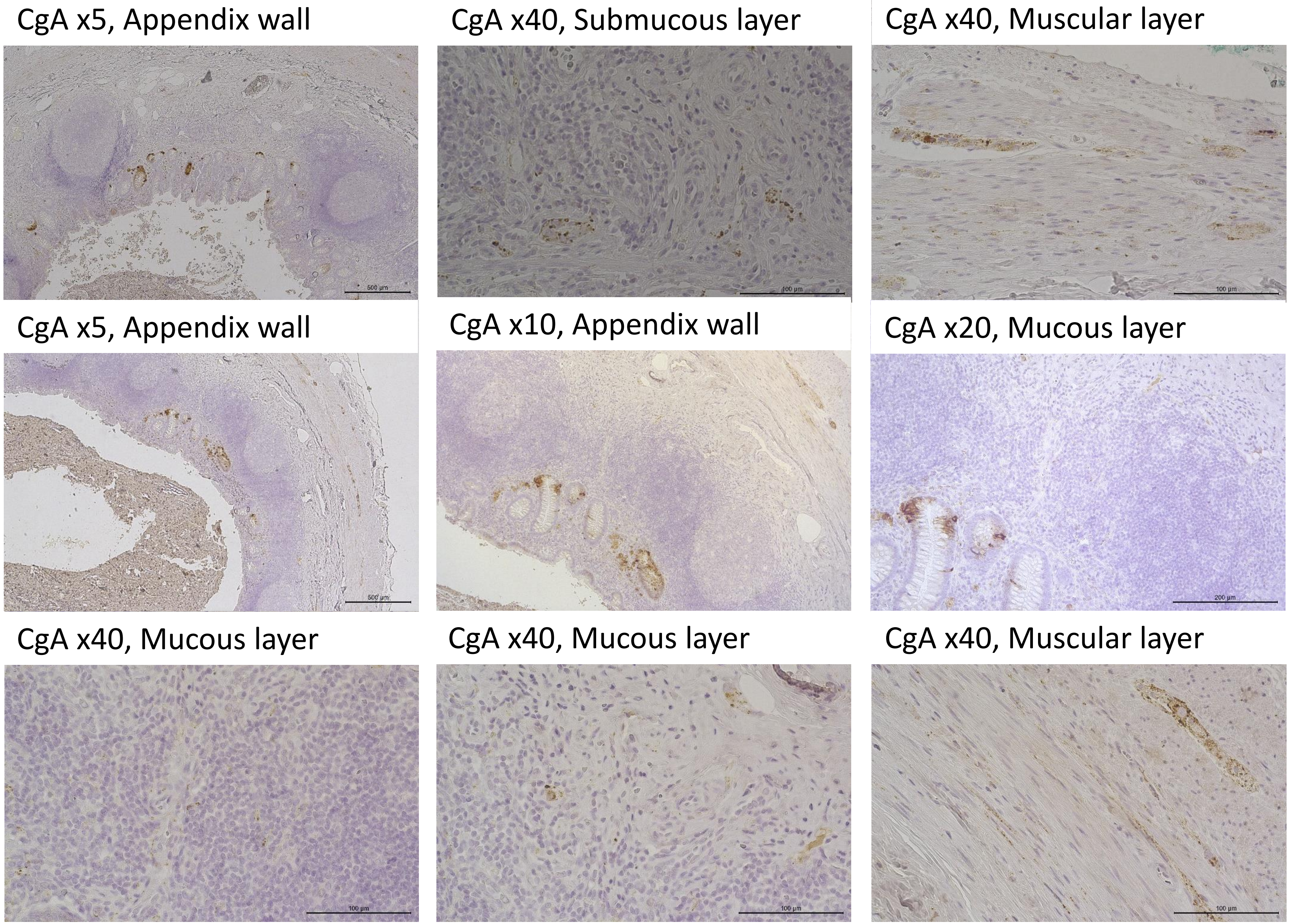

Supplementary data: Representative figures of patient’s diagnostic panel. Sample numbers have been coded.

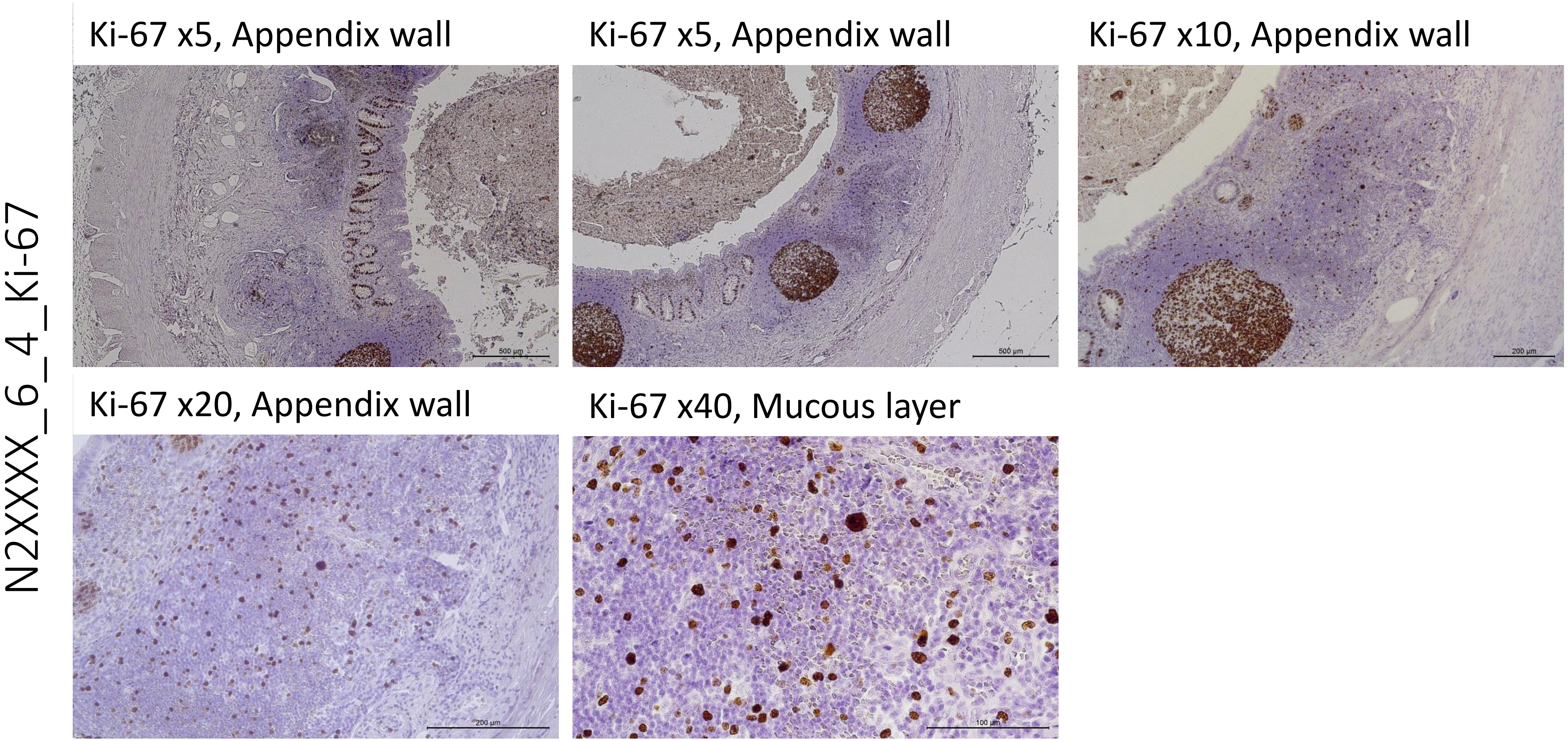

Supplementary data: Representative figures of patient's diagnostic panel. Sample numbers have been coded. Magnification of the lens is given, Leica DM5000B, Wetzlar, Germany.

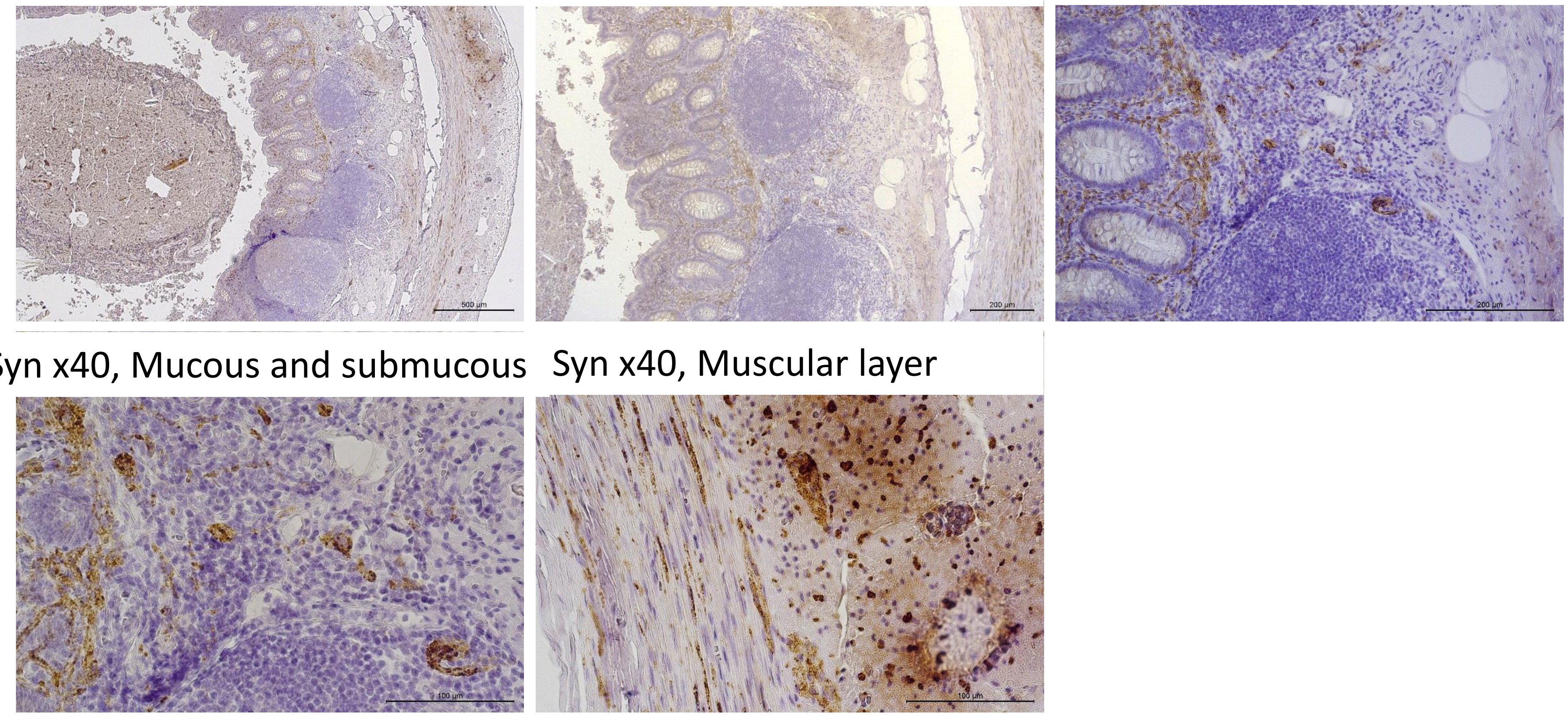

Supplementary data: Representative figures of patient's diagnostic panel. Sample numbers have been coded. Magnification of the lens is given, Leica DM5000B, Wetzlar, Germany.

N5XXXX_6_3_Ki67

Ki67 x5, Appendix wall

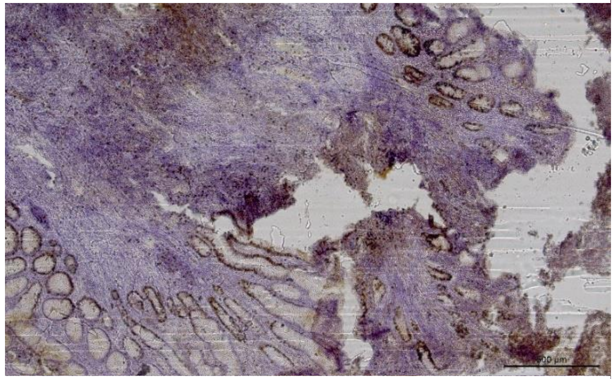

Ki67 x5, Appendix wall

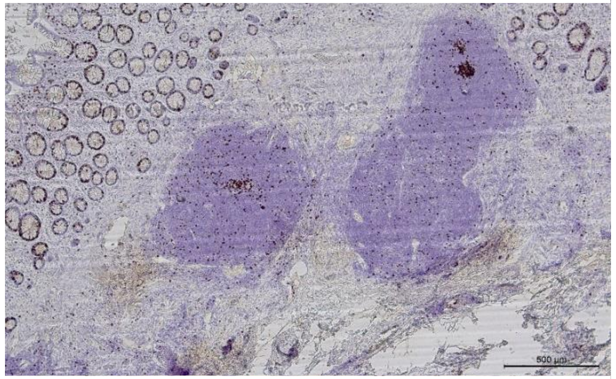

Ki67 x5, Appendix wall

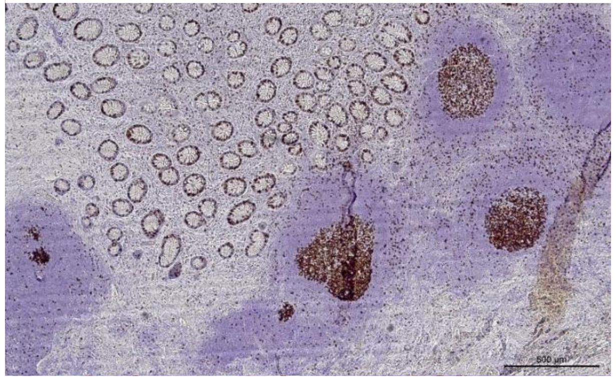

Ki67 x5, Appendix wall

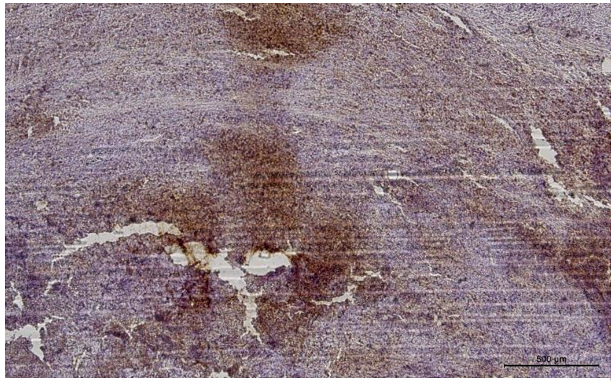

Ki67 x10, Appendix wall

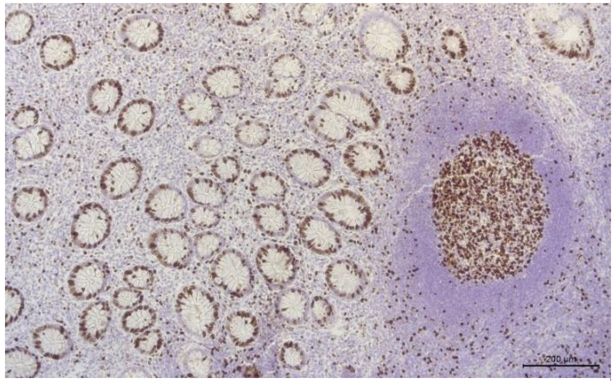

Ki67 x10, Appendix wall

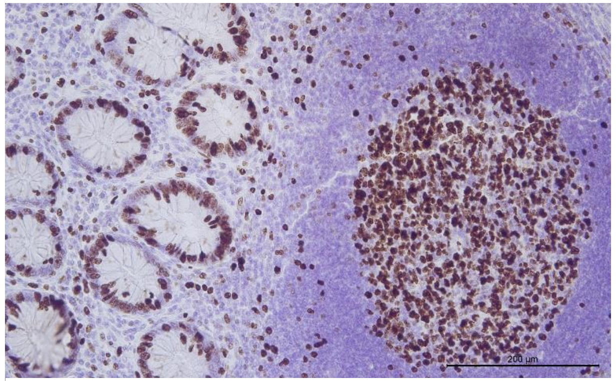

Ki67 x10, Appendix wall

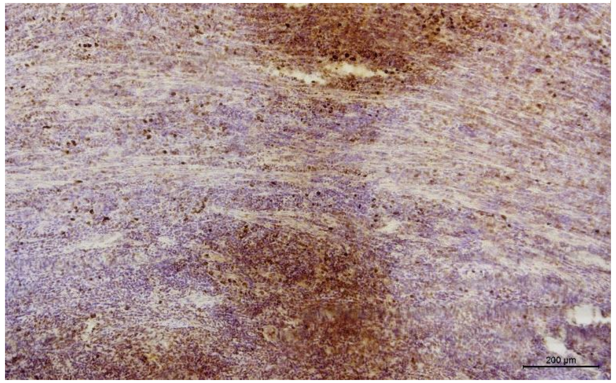

Ki67 x20, Appendix wall

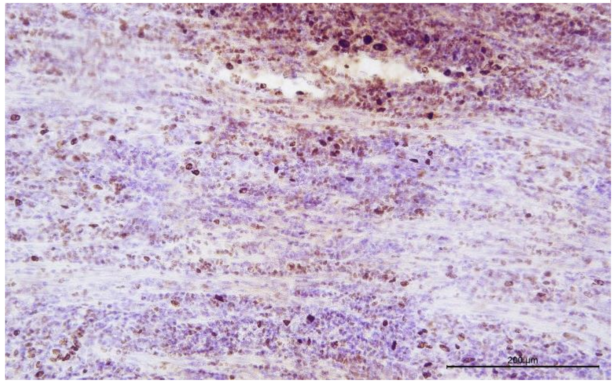

Ki67 x40, Mucous layer

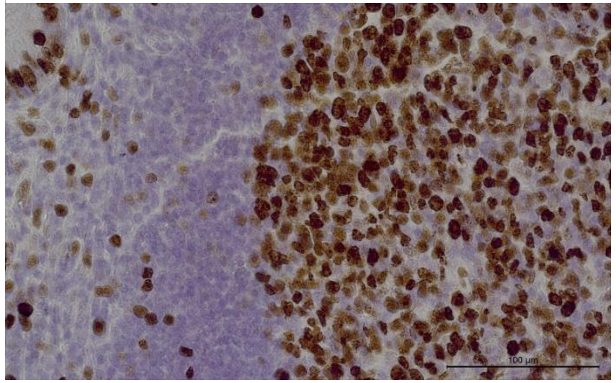

Supplementary data: Representative figures of patient's diagnostic panel. Sample numbers have been coded.

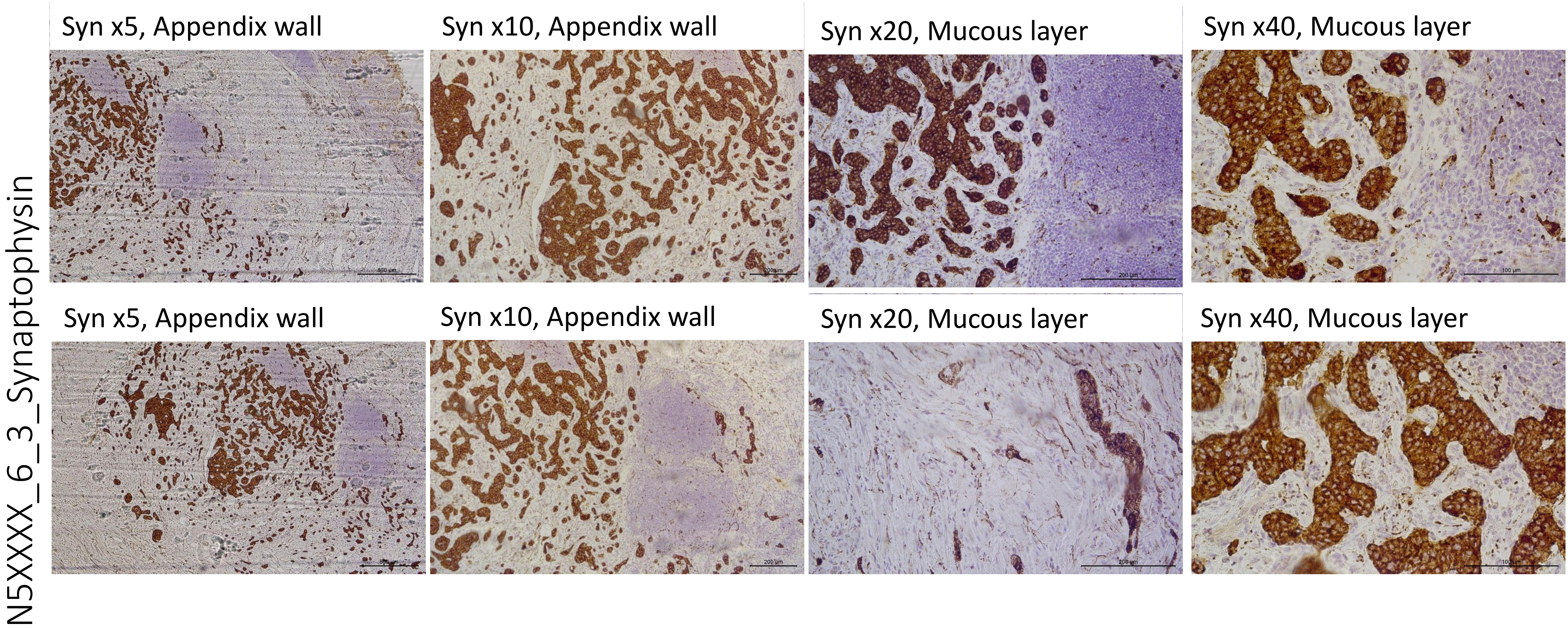

Supplementary data: Representative figures of patient's diagnostic panel. Sample numbers have been coded.
Magnification of the lens is given, Leica DM5000B, Wetzlar, Germany.

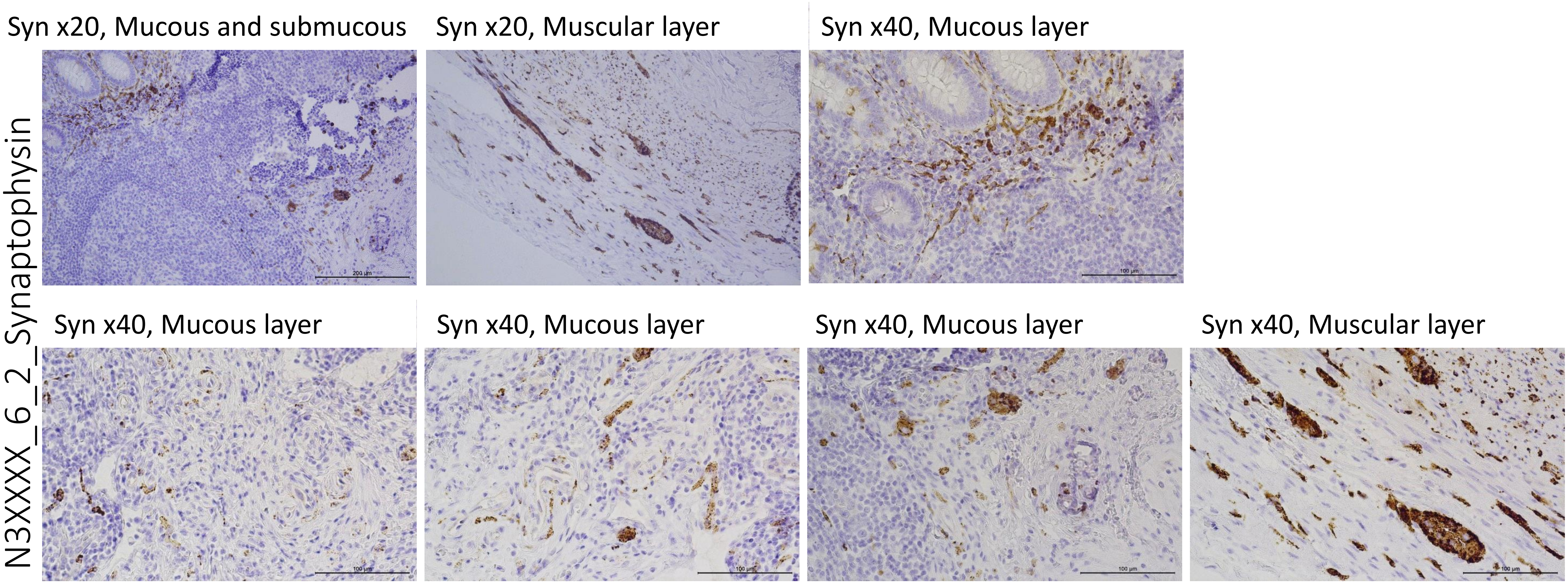

Supplementary data: Representative figures of patient's diagnostic panel. Sample numbers have been coded. Magnification of the lens is given, Leica DM5000B, Wetzlar, Germany.

N3XXXX_6_2_Chromogranin A

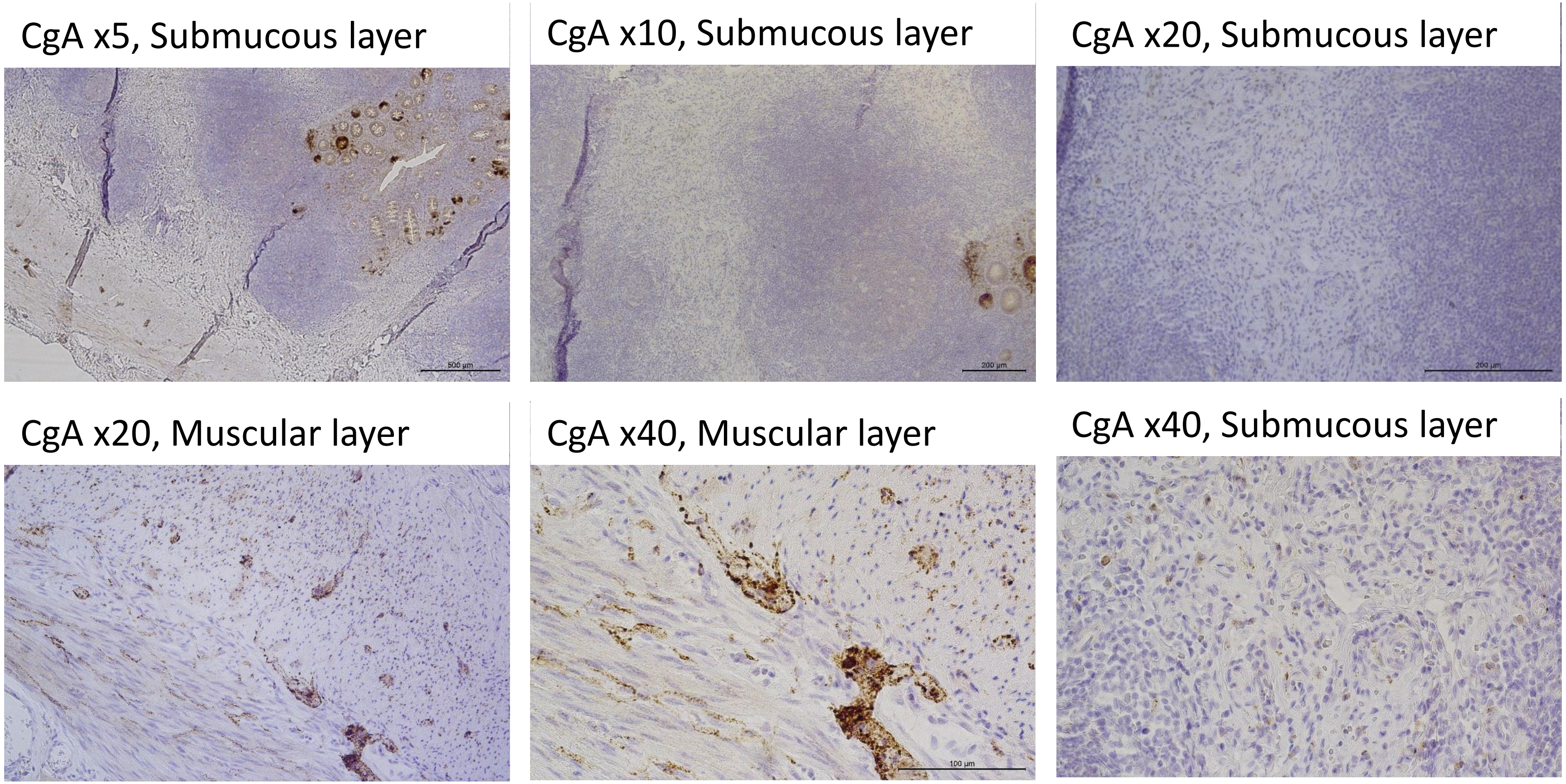

Supplementary data: Representative figures of patient’s diagnostic panel. Sample numbers have been coded. Magnification of the lens is given, Leica DM5000B, Wetzlar, Germany.

N3XXXX_6_2_Ki-67

Ki-67 x5, Appendix wall | Ki-67 x10, Appendix wall | Ki-67 x20, Submucous layer

Ki-67 x5, Appendix wall | Ki-67 x20, Submucous layer | Ki-67 x20, Muscular layer

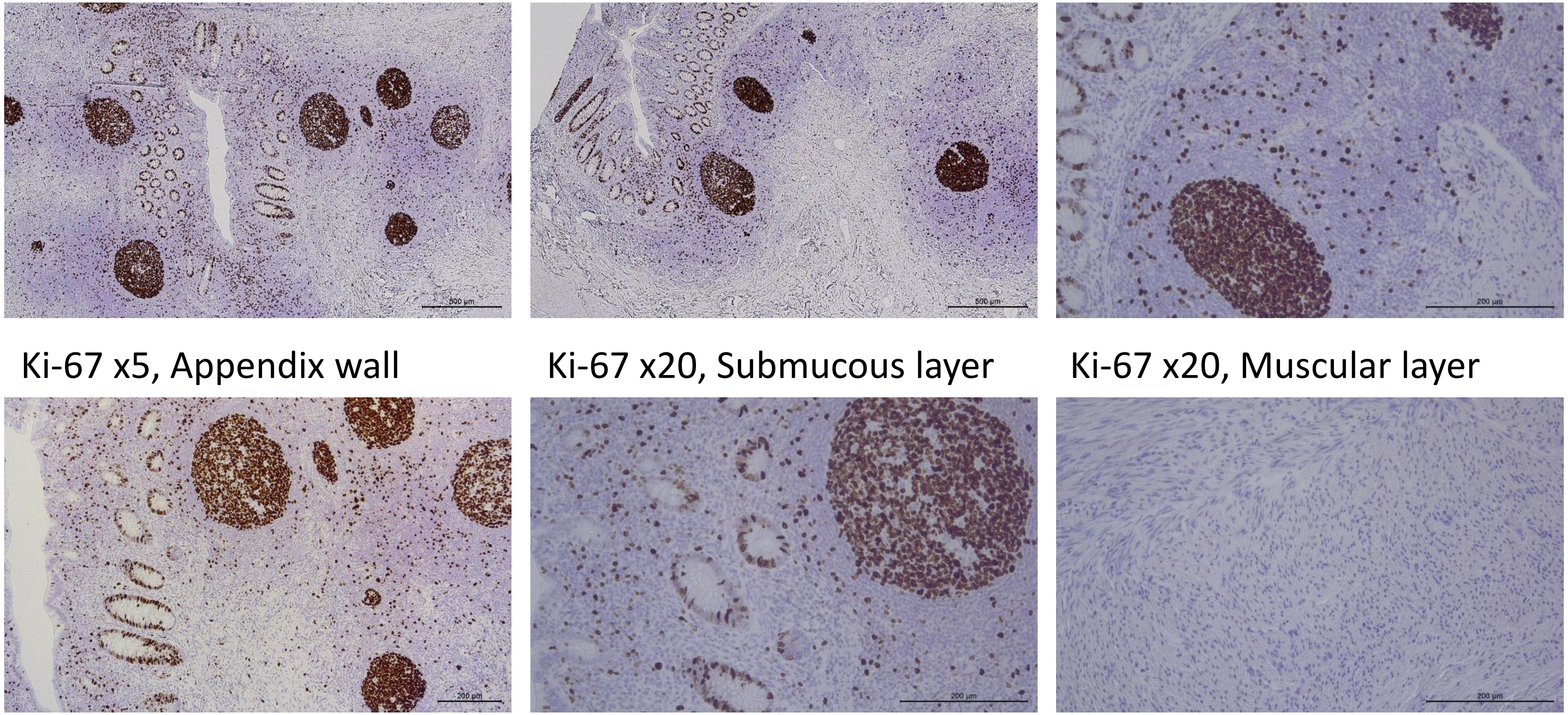

Supplementary data: Representative figures of patient’s diagnostic panel. Sample numbers have been coded. Magnification of the lens is given, Leica DM5000B, Wetzlar, Germany.
