# Supplementary figures and images for: Pilot Study: FSHR Expression in Neuroendocrine Tumors of the Appendix
Source: J Clin Med. 2023 Aug 2;12(15):5086. doi: 10.3390/jcm12155086 (PMC10419379; doi:10.3390/jcm12155086)

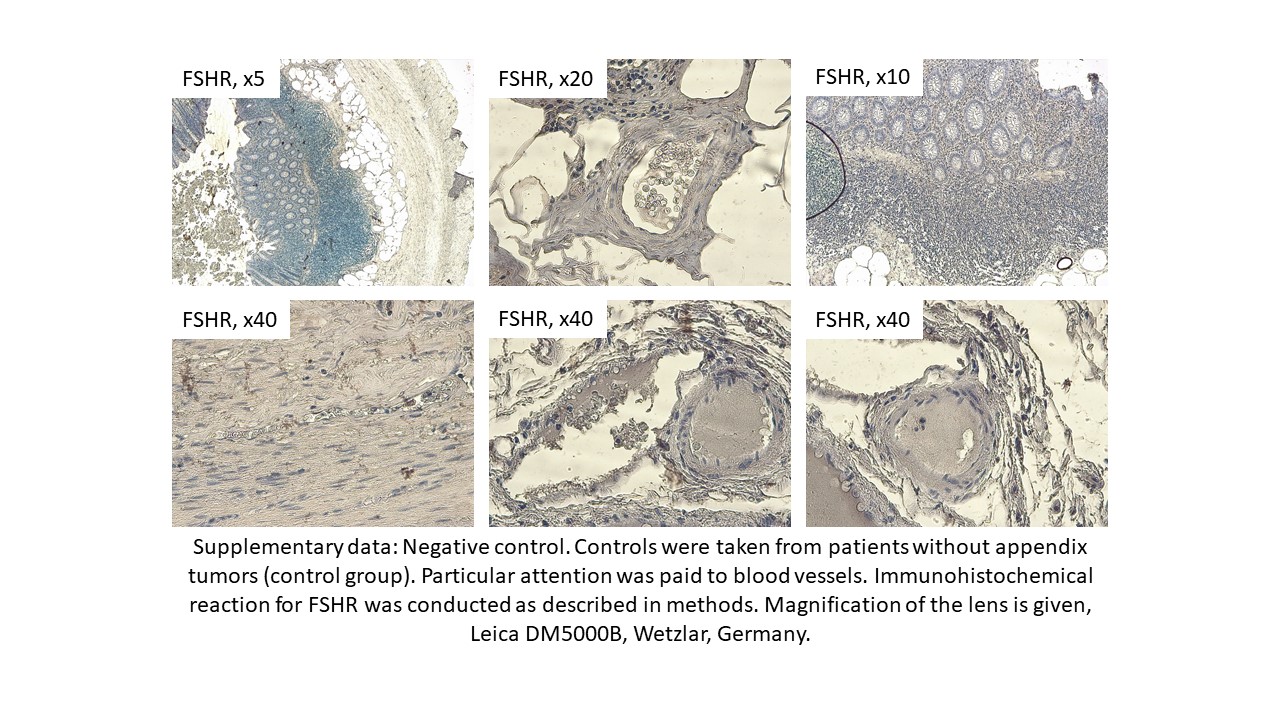

Supplement: Supplementary file 1 [file jcm-12-05086-s001.zip › Figura negative supplementray data.JPG]
